# Destruction of Full-Length Androgen Receptor by Wild-Type SPOP, but Not Prostate-Cancer-Associated Mutants

**DOI:** 10.1016/j.celrep.2014.01.013

**Published:** 2014-02-06

**Authors:** Jian An, Chenji Wang, Yibin Deng, Long Yu, Haojie Huang

**Affiliations:** 1 State Key Laboratory of Genetic Engineering, Institute of Genetics, Fudan University, Shanghai 200433, China; 2 Department of Biochemistry and Molecular Biology, Mayo Clinic College of Medicine, Rochester, MN 55905, USA; 3 Laboratory of Cancer Genetics, The University of Minnesota Hormel Institute, Austin, MN 55912, USA; 4 Institutes of Biomedical Sciences, Fudan University, Shanghai 200433, China; 5 Department of Urology, Mayo Clinic College of Medicine, Rochester, MN 55905, USA; 6 Mayo Clinic Cancer Center, Mayo Clinic College of Medicine, Rochester, MN 55905, USA; 7 These authors contributed equally to this work

## Abstract

The *SPOP* E3 ubiquitin ligase gene is frequently mutated in human prostate cancers. Here, we demonstrate that SPOP recognizes a Ser/Thr-rich degron in the hinge domain of androgen receptor (AR)and induces degradation of full-length AR and inhibition of AR-mediated gene transcription and prostate cancer cell growth. AR splicing variants, most of which lack the hinge domain, escape SPOP-mediated degradation. Prostate-cancer-associated mutants of SPOP cannot bind to and promote AR destruction. Furthermore, androgens antagonize SPOP-mediated degradation of AR, whereas antiandrogens promote this process. This study identifies AR as a bona fide substrate of SPOP and elucidates a role of SPOP mutations in prostate cancer, thus implying the importance of this pathway in resistance to antiandrogen therapy of prostate cancer.

## INTRODUCTION

Prostate cancer is one of the most common malignancies, with over 913,000 new cases and over 261,000 deaths worldwide each year (Ferlay et al., 2010). Although androgen deprivation therapies are initially effective in approximately 90% of prostate cancer patients, the disease inevitably recurs as lethal castration-resistant prostate cancer (CRPC).

Androgen receptor (AR) is a pivotal transcription factor that is essential for normal prostate cell growth and survival. AR is also important for initiation and progression of prostate cancer. The role of AR in prostate cancer initiation is accentuated by the seminal discovery that the oncogenic ETS family transcription factors, such as ERG and ETV1, are translocated to the loci of androgen regulated genes including *TMPRSS2* in approximately 50% of all human prostate cancers (Kumar-Sinha et al., 2008; Tomlins et al., 2005). Development of CRPC is considered to be causally related to a persistent activation of AR by a number of mechanisms, including, but not limited to, AR amplification or overexpression; gain-of-function mutations that allow AR to be activated by other steroids or antiandrogens; ligand-indepen-dent activation of the AR by cytokine/growth factor-dependent pathways; overexpression of AR coactivators; intracrine signaling by increased intratumoral androgen synthesis; and expression of constitutively active splicing variants of AR (Cai et al., 2011; Chen et al., 2004; Dehm and Tindall, 2011; Grossmann et al., 2001; Scher and Sawyers, 2005). The importance of AR reactivation during castration-resistant progression of prostate cancer has been clinically confirmed by the effective treatment of CRPC by second-generation androgen-AR axis inhibitors including abiraterone and enzalutamide (MDV3100) (de Bono et al., 2011; Scher et al., 2012).

Covalent attachment of ubiquitin via enzyme cascades (E1, E2s, and E3s) constitutes a fundamental mechanism that promotes either protein turnover or signaling transduction. Ubiquitin ligases, or E3s, selectively bind to and target substrates for ubiq-uitination and subsequent proteasome degradation. The largest E3 ligase subfamily consists of Cullin-RING ligases (CRLs), which are multisubunit enzymes, consisting of hundreds of distinct CRL complexes with the capacity to recruit numerous substrates (Petroski and Deshaies, 2005). Human cells express seven different CULLINs (CUL1, 2, 3, 4A, 4B, 5, and 7), each of which nucleates a multisubunit E3 ubiquitin ligase complex (Petroski and Deshaies, 2005). The CRL3 complex is composed of the scaffold CUL3 and RING protein RBX1, in combination with a BTB (Bric-a-brac/Tramtrack/Broad complex) domain protein that acts as an adaptor for substrate binding. The human genome encodes more than 180 BTB proteins. One well-characterized BTB protein is SPOP, which contains a substrate-binding MATH domain at the N-terminal and a CUL3-binding BTB domain at the C-terminal. SPOP has been linked to ubiquitination of several substrates in *Drosophila* and human, such as Puc, Ci/Gli, MacroH2A, Daxx, and SRC-3 (Hernández-Muñoz et al., 2005; Kwon et al., 2006; Li et al., 2011; Liu et al., 2009; Zhang et al., 2006).

Mounting evidence indicates that dysregulation of the ubiqui-tin-proteasome pathway is involved in cancer pathogenesis. Systematic whole-genome or exome sequencing of prostate tumors has led to the identification of frequent somatic mutations in *SPOP* (Barbieri et al., 2012; Berger et al., 2011; Grasso et al., 2012; Kan et al., 2010). Interestingly, all SPOP mutations described thus far affect evolutionarily conserved residues in the structurally defined substrate-binding MATH domain. Importantly, prostate tumors that contain mutated *SPOP* almost completely lack mutations in *PTEN* and *TP53* tumor suppressors, suggesting a new molecular subtype of prostate cancer (Barbieri et al., 2012). In addition to mutations, SPOP protein expression is often downregulated in prostate tumors (Kim et al., 2013). However, how this contributes to prostate cancer pathogenesis and progression remains to be defined. In this study, we identified AR as a degradation substrate of SPOP in prostate cancer cells.

## RESULTS

### AR Is a Bona Fide Substrate of the SPOP-CUL3-RBX1 E3 Ligase Complex

**Figure 1:**
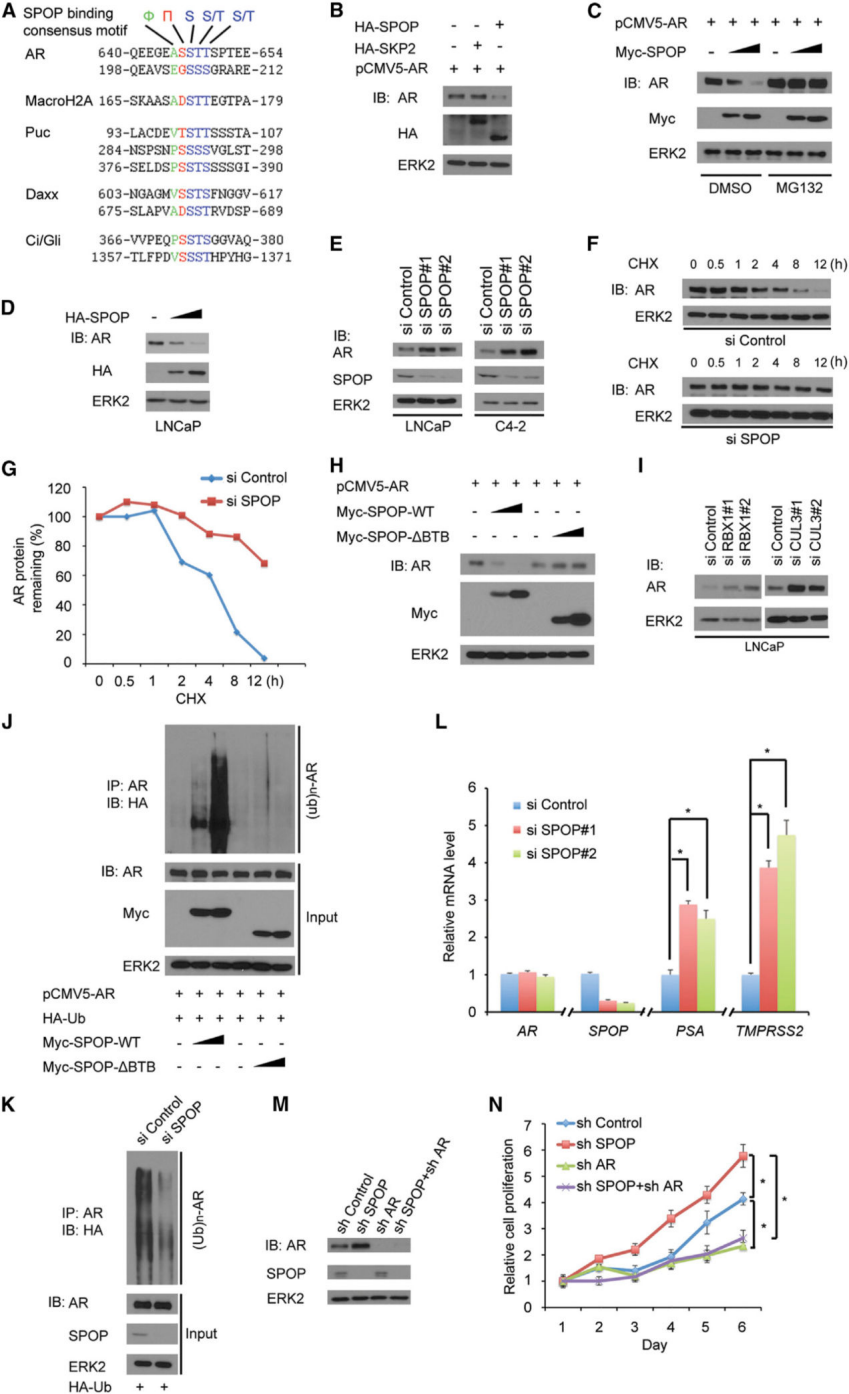
The SPOP-CUL3-RBX1 Complex Targets AR for Ubiquitination and Degradation (A) Comparison of putative SPOP binding sites in AR with the SPOP-binding consensus motif defined in the known SPOP substrates. (B) Ectopically expressed SPOP promotes exogenous AR protein degradation. 293T cells were transfected with indicated constructs for 24 hr followed by western blot (WB). ERK2, a loading control. (C) SPOP regulates AR protein levels through the proteasome pathway. 293T cells were transfected with 4 µg pCMV5-AR and 0, 2, or 4 µg Myc-SPOP plasmids. After 16 hr, cells were treated with 20 µM MG132 for 8 hr and harvested for WB. (D) SPOP regulates endogenous AR protein levels. LNCaP cells were transfected with 0, 2,or4 µgHA-SPOP plasmid for 24 hr before cells were harvested for WB. (E) Knockdown of SPOP increases endogenous AR protein levels. LNCaP and C4-2 cells were transfected with control or two independent SPOP-specific siRNA. After 48 hr, cells were harvested for WB. (F and G) SPOP knockdown prolongs AR protein half-life. LNCaP cells were transfected with control or SPOP-specific siRNA. After 48 hr, cells were treated with 50 µg/µl cycloheximide (CHX). At different time points, cells were harvested for WB (F). At each time point, the intensity of AR was normalized to the intensity of ERK2 (loading control) first and then to the value at the 0 hr time point (G). A similar result was obtained in two independent experiments. (H) The BTB domain in SPOP is essential for SPOP-mediated degradation of AR. We transfected 4 µg pCMV5-AR and 0, 2, or 6 µg Myc-SPOP-WT or Myc-SPOP-ΔBTB plasmids into 293T cells for 24 hr, followed by WB. (I) Knockdown of RBX1 or CUL3 increases endogenous AR protein levels. LNCaP cells were transfected with control siRNA or siRNAs for RBX1 or CUL3 for 48 hr followed by WB. (J) SPOP promotes AR polyubiquitination in vivo. 293T cells were transfected with indicated plasmids for 16 hr followed by treatment with 20 µM MG132 for 8 hr. Immunoprecipitated AR proteins were analyzed by WB for ubiquitination. (K) Knockdown of SPOP decreases ubiquitination of endogenous AR. LNCaP cells were transfected with indicated plasmids and siRNAs for 40 hr followed by treatment with 20 µM MG132 for 8 hr, and then the same procedure was performed as shown in (J). (L) Quantitative RT-PCR measurement of the mRNA level of *SPOP, AR*, and two AR target genes (*PSA* and *TMPRSS2*) in SPOP-knockdown LNCaP cells. The mRNA level of *GAPDH* was used for normalization. All data shown are mean values ± SD (error bar) from three independent experiments. p < 0.01. (M and N) SPOP inhibits prostate cancer cell growth via regulation of AR. C4-2 cells were infected with lentivirus expressing control or SPOP and/or AR-specific small hairpin RNAs. Forty-eight hours after infection, cells were harvested for WB (M) or cultured in androgen-depleted medium for MTS assay (N). All data shown are mean values ± SD (error bar) from six replicates. p < 0.01.

All SPOP mutations detected thus far in prostate cancer occur in the structurally defined substrate-binding motif (Barbieri et al., 2012; Zhuang et al., 2009), suggesting that the pathophysiology of SPOP mutations in prostate cancer is relevant to its function in substrate binding and degradation. A SPOP-binding consensus motif Φ-π-S-S/T-S/T (Φ: nonpolar residues, π: polar residues) has been identified in several SPOP substrates including Puc, MacroH2A, Ci/Gli, and Daxx (Hernández-Muñoz et al., 2005; Kwon et al., 2006; Liu et al., 2009; Zhang et al., 2006). We performed a protein motif search and discovered that AR harbors one perfectly matched (^645^ASSTT^649^) and one imperfectly matched (^203^EGSSS^207^) SPOP-binding motif (Figure 1A). This observation prompted us to investigate whether SPOP functions as an E3 ubiquitin ligase of AR. As shown in Figure 1B, coexpression of SPOP, but not the related E3 ligase SKP2, decreased the ectopically expressed AR protein level in 293T cells. This effect was completely blocked by treatment with the proteasome inhibitor MG132 (Figure 1C), indicating that SPOP downregulates AR protein levels via the proteasome pathway.

Next, we examined the effect of SPOP on degradation of endogenous AR. Overexpression of SPOP in LNCaP prostate cancer cells resulted in a marked reduction of the endogenous level of AR protein in a dose-dependent manner (Figure 1D). Knockdown of endogenous SPOP by two independent SPOP-specific small interfering RNAs (siRNAs) increased AR protein levels in both LNCaP and C4-2 prostate cancer cells (Figure 1E). Furthermore, knockdown of SPOP markedly prolonged the half-life of endogenous AR protein in LNCaP cells (Figures 1F and 1G).

SPOPisa substrate-binding subunit of the SPOP-CUL3-RBX1 E3 ligase complex. The C-terminal BTB domain of SPOP is essential for its interaction with the scaffold protein CULLIN3 (CUL3). The BTB deletion mutant of SPOP (SPOP-ΔBTB) cannot form a complex with CUL3 and RBX1 to function as an enzymatically active E3 ligase (Weber et al., 2005). As shown in Figure 1H, only wild-type SPOP, but not the SPOP-ΔBTB mutant, promoted AR degradation, indicating that the BTB domain and the CUL3-RBX1 complex are required for SPOP-mediated degradation of AR. Next, we sought to determine whether other subunits of the SPOP-CUL3-RBX1 E3 ligase complex are required for AR degradation. We knocked down RBX1 or CUL3 by two independent gene-specific siRNAs and examined AR protein levels in LNCaP cells. As shown in Figure 1I, knockdown of either RBX1 or CUL3 resulted in a dramatic increase in AR protein levels. These data suggest that the E3 ligase function of SPOP is required for its destruction of AR.

To determine whether SPOP regulates AR polyubiquitination, HA-Ub and pCMV5-AR were coexpressed in 293T cells with different doses of wild-type SPOP (SPOP WT) or enzymatic dead mutant (SPOP-ΔBTB). AR protein was robustly polyubi-quitinated by coexpression of SPOP WT, but not SPOP-ΔBTB, in a dose-dependent manner (Figure 1J). Accordingly, knockdown of endogenous SPOP in LNCaP cells decreased the polyubiquitination of endogenous AR (Figure 1K). These data support the concept that the SPOP regulates AR stability through ubiquitin-dependent proteasome degradation in prostate cancer cells.

Consistent with the finding that SPOP knockdown increased AR protein levels (Figure 1E), it also increased the transcriptional activity of AR by upregulating mRNA expression of *PSA* and *TMPRSS2*, two well-studied AR transcriptional targets (Figure 1L). In contrast, SPOP knockdown had no effect on expression of *AR* mRNA itself, further indicating that the effect of SPOP on AR is not mediated by regulation of *AR* expression at the mRNA level. In agreement with a previous report that overex-pression of wild-type SPOP inhibits the growth of LNCaP-Abl CRPC cells (Geng et al., 2013), knockdown of endogenous SPOP increased the growth of C4-2 CRPC cells (Figures 1M and 1N). Importantly, this effect was almost completely abrogated by concomitant knockdown of AR (Figure 1N). Moreover, AR knockdown alone markedly inhibited C4-2 cell growth (Figure 1N), which is in agreement with a previous report (Zegarra-Moro et al., 2002). Together, these findings suggest that SPOP promotes AR protein ubiquitination and proteasome degradation and inhibits prostate cancer cell growth in an AR-dependent manner.

### SPOP Interacts with AR In Vitro and In Vivo

**Figure 2:**
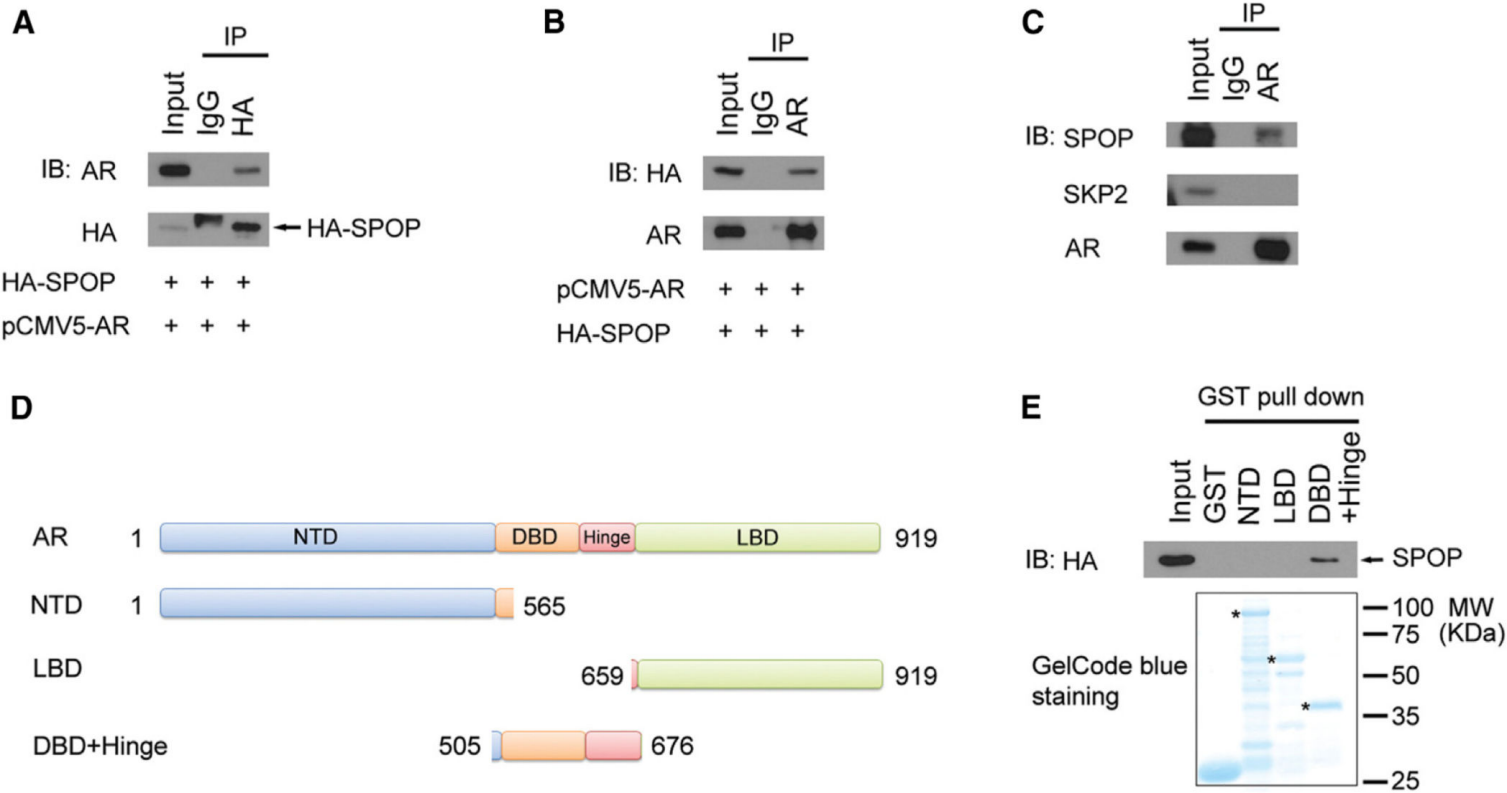
SPOP Interacts with AR In Vitro and In Vivo (A and B) Ectopically expressed SPOP and AR proteins interact with each other. 293T cells were transfected with indicated plasmids for 16 hr followed by treatment with 20 µM MG132 for 8 hr and coIP with anti-HA (A) or anti-AR (B) and WB. (C) Endogenous SPOP and AR proteins interact with each other in LNCaP cells. After treatment with 20 µM MG 132 for8 hr, cell lysates were prepared for coIP with AR antibody and WB. SKP2 was included as a negative control. (D) Schematic diagram of three GST-AR recombinant proteins. NTD, NH2-terminal domain; DBD, DNA binding domain; LBD, ligand binding domain. (E) SPOP binds to the central part of AR including DBD and the hinge domain. Bacterially expressed GST fusion proteins of NTD, LBD, and DBD plus the hinge domain were incubated with cell lysates of 293T cells transfected with HA-SPOP and subjected to GST pull-down assay. Bound HA-SPOP was detected by WB with HA antibody, and GST fusion proteins were detected by GelCode blue staining.

Because substrate binding is a key event for E3 ligase-mediated ubiquitination and subsequent proteasome degradation, we examined the binding of AR by SPOP using coimmunoprecipitation (coIP) assay. As shown in Figure 2A, ectopically expressed AR protein was coimmunoprecipitated by HA-SPOP. A similar result was obtained in a reciprocal coIP experiment using AR antibody (Figure 2B). Importantly, endogenous SPOP and AR proteins were present in the same complex coimmunoprecipitated by AR antibody in LNCaP cells (Figure 2C). Next, we sought to determine which domain(s) of AR is required for SPOP binding. AR has four well-defined functional domains including the N-terminal domain (NTD), DNA binding domain (DBD), hinge domain, and ligand binding domain (LBD) (Figure 2D). We purified glutathione S-transferase (GST) recombinant proteins for three truncation mutants of AR: NTD (amino acids 1–565), DBD plus hinge domain (amino acids 505–676), and LBD (amino acids 659–919). The GST pull-down assay demonstrated that SPOP binds specifically to the central region of AR including the DBD and hinge domain, but not the AR NTD and LBD, or GST alone (Figure 2E).

### The ^645^ASSTT^649^ Motif in the AR hinge Domain Is Required for SPOP-Mediated Degradation of AR

**Figure 3:**
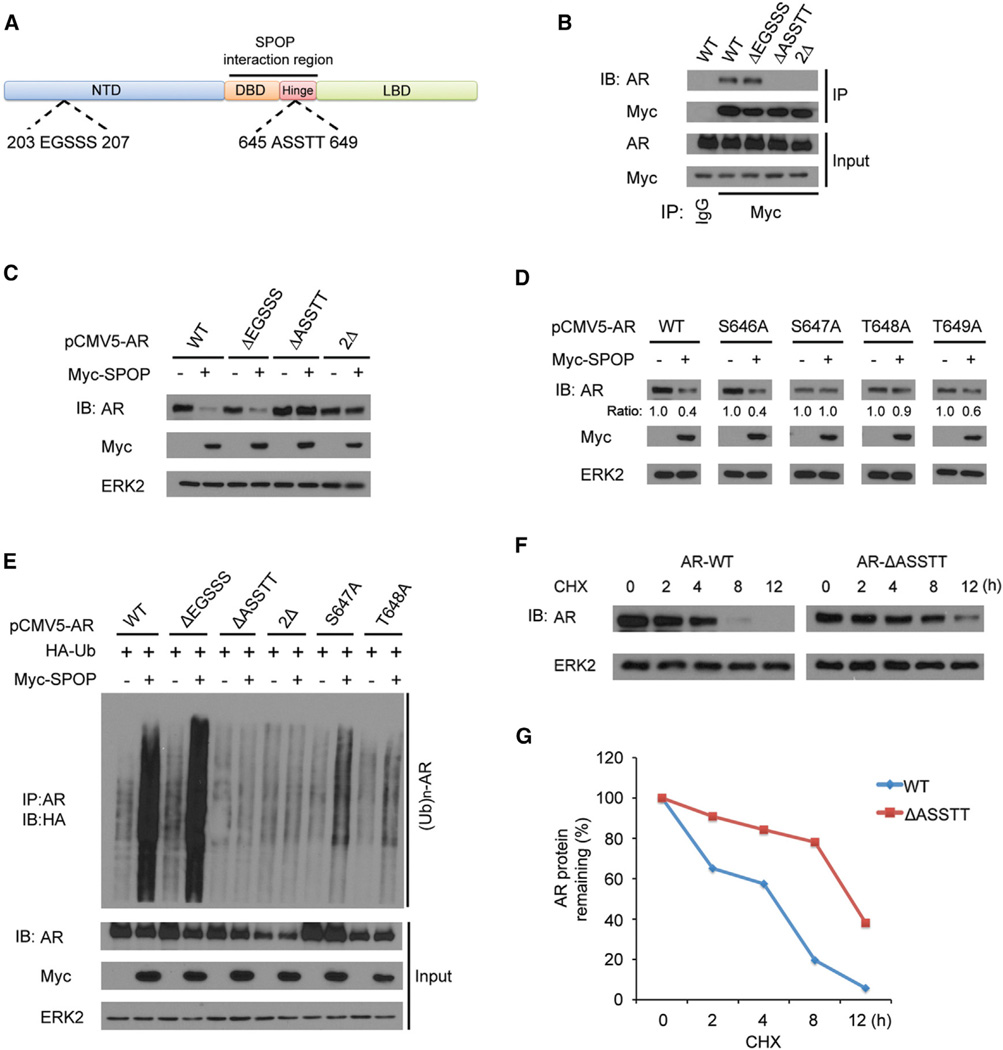
The ^645^ASSTT^649^ Motif in AR Is a Degron Recognized by SPOP (A) Diagram showing two putative SPOP-binding motifs located in AR NTD and the hinge domain, respectively. (B) The ^645^ASSTT^649^ motif is required for AR binding to SPOP. 293T cells were transfected with plasmids for Myc-SPOP and wild-type (WT) and three deletion mutants of AR(ΔEGSSS, ΔASSTT, and double deletion 2Δ). After16hr, cells were treated with 20 µM MG132 for8 hr followed by coIP with Myc antibody and WB. (C) The ^645^ASSTT^649^ motif is required for AR degradation by SPOP. 293T cells were transfected with indicated plasmids for 24 hr followed by WB. (D) S647 and T648 residues in the ^645^ASSTT^649^ motif are critical for SPOP-induced degradation of AR. 293T cells were transfected with the indicated plasmids for 24 hr followed by WB. The density of AR was determined by normalizing to ERK2 (loading control) first and then to the normalized value in Myc-SPOP-untransfected cells. (E) The ^645^ASSTT^649^ motif is essential for SPOP-induced AR polyubiquitination. 293T cells were transfected with the indicated plasmids for 16 hr followed by treatment with 20 µM MG132 for 8 hr, IP, and WB. (F and G) Deletion of the ^645^ASSTT^649^ motif prolongs the half-life of AR protein. AR WT or AR ΔASSTT mutant was transfected into 293T cells for 24 hr followed by treatment with 50 µg/µl cycloheximide (CHX). At various time points, cells were harvested for WB (F). At each time point, the intensity of AR was normalized to the intensity of ERK2 first and then to the value at the 0 hr time point (G). A similar result was obtained in two independent experiments.

As mentioned above, AR harbors one perfectly matched (^645^ASSTT^649^) and one imperfectly matched (^203^EGSSS^207^) SPOP-binding motif (Figure 1A). Because^645^ASSTT^649^ is located in the SPOP interaction region identified by GST pull-down assay (Figure 3A), we sought to determine if the ^645^ASSTT^649^ motif plays a role in regulation of SPOP-AR interaction. To this end, we generated three AR deletion mutants, in which the ^203^EGSSS^207^ motif (in the NTD) and the ^645^ASSTT^649^ motif were deleted individually or together. CoIP assays demonstrated that AR-WT and the ΔEGSSS mutant were coimmunoprecipitated by Myc-SPOP. In contrast, ΔASSTT and the double mutant (2Δ) totally lost the SPOP binding capability (Figure 3B). These data indicate that the perfectly matched SPOP-binding consensus ^645^ASSTT^649^ motif, but not the imperfectly matched ^203^EGSSS^207^ motif, is required for SPOP-AR interaction. Next, we determined if the ^645^ASSTT^649^ motif is essential for SPOP-mediated AR degradation. As shown in Figure 3C, SPOP efficiently targeted both AR-WT and the ΔEGSSS mutant for degradation, but not the ΔASSTT and 2Δ mutants. These results suggest that the ^645^ASSTT^649^ motif located in the hinge domain, but not the ^203^EGSSS^207^ motif in NTD, is critical for AR binding by SPOP and subsequent degradation. To further investigate if the serine and/or threonine residues in the ^645^ASSTT^649^ motif are crucial for AR binding by SPOP, we generated four point mutants of AR (S646A, S647A, T648A, and T649A) to determine how SPOP regulates degradation of these mutants. Overexpression of SPOP decreased the protein level of AR-WT, S646A, and T649A mutants, but had little or no effect on the level of AR-S647A or T648A mutants (Figure 3D). To further determine the importance of the ^645^ASSTT^649^ motif as a degron, AR WT or mutants (ΔEGSSS, ΔASSTT, 2Δ, S647A, and T648A) were cotrans-fected with or without SPOP in 293T cells. In vivo, ubiquitination assays demonstrated that SPOP robustly enhanced polyubiquitination of AR-WT and the ΔEGSSS mutant, but not the ΔASSTT and 2Δ mutants (Figure 3E). Consistent with protein degradation, S647A and S648A mutations largely diminished SPOP-induced AR polyubiquitination. Furthermore, the ΔASSTT mutation prolonged the half-life of AR protein (Figures 3F and 3G). Thus, these data demonstrate that the ^645^ASSTT^649^ motif functions as an AR degron, which is essential for SPOP binding and subsequent ubiquitin-dependent degradation of AR, and that Ser647 and Thr648 residues within this motif are crucial for this function.

### Prostate Cancer-Derived Hinge Domain-Deficient AR Splicing Variants Escape SPOP-Mediated Proteasome Degradation

**Figure 4:**
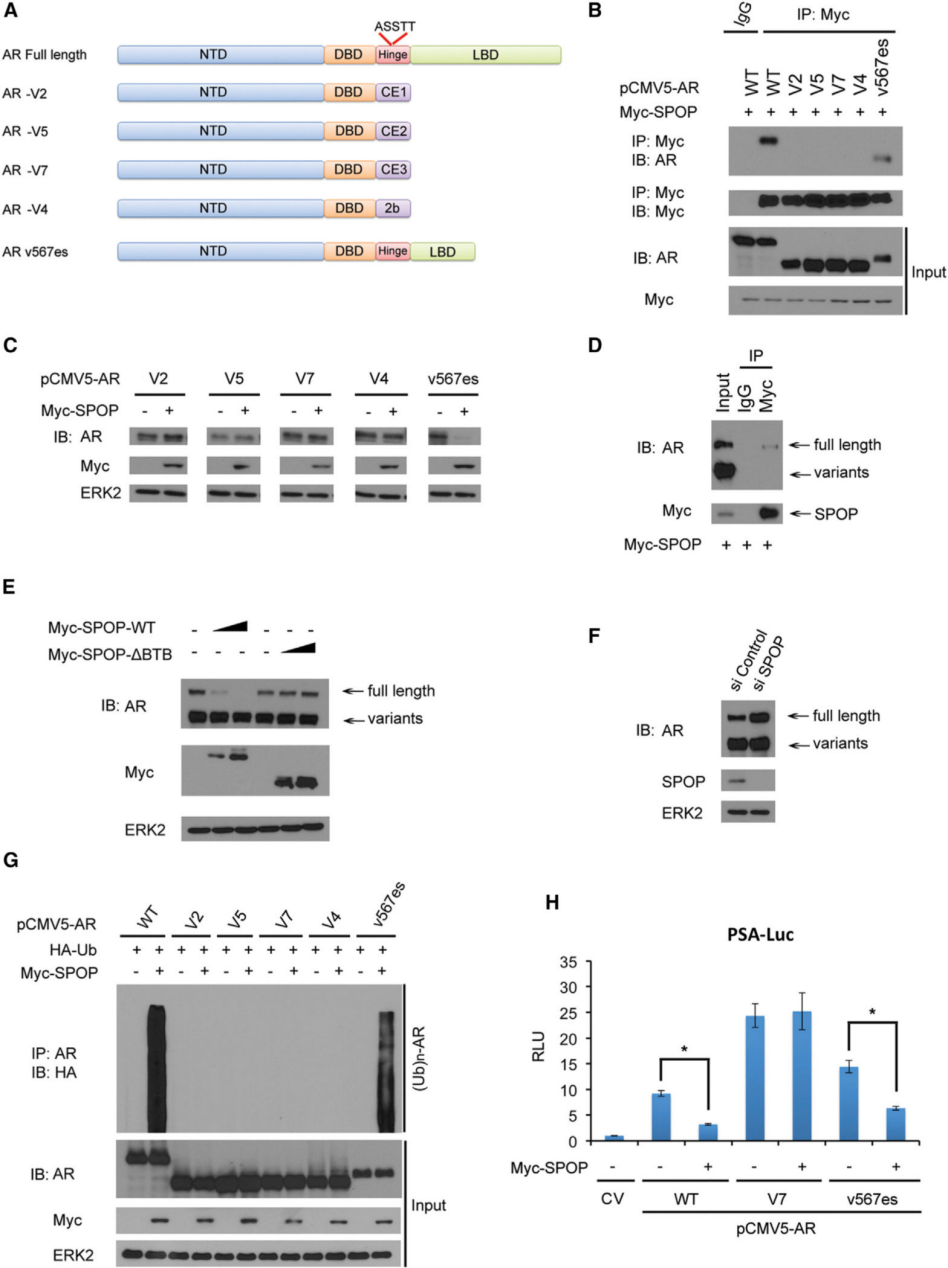
Hinge Domain Null AR Splicing Variants Are Resistant to SPOP-Mediated Degradation (A) Schematic diagram of full-length AR and five AR splicing variants (V2, V5, V7, V4, and v567es). Only full-length AR and v567es contain the ^645^ASSTT^649^ motif. (B) Hinge domain-deficient AR splicing variants lose the capacity of binding to SPOP. 293T cells were transfected with the indicated plasmids for 16 hr followed by treatment with 20 µM MG132 for 8 hr, coIP, and WB. (C) Hinge domain null AR splicing variants are resistant to SPOP-promoted degradation. 293T cells were transfected with the indicated plasmids for24 hr followed by WB. (D) SPOP binds to endogenous full-length AR but not the variants in 22Rv1 cells. Cells were transfected with Myc-SPOP. After 16 hr, cells were treated with MG132 for 8 hr. Cell lysates were subjected to coP and WB. (E) Ectopically expressed SPOP differentially targets endogenous full-length AR and variants for degradation in 22Rv1 cells. Cells were transfected with 0, 2, or 4 µg plasmid for Myc-SPOP-WT or Myc-SPOP-ΔBTB. After 24 hr, cell lysates were prepared for WB. (F) Knockdown of endogenous SPOP increases protein levels of endogenous full-length AR but not variants in 22Rv1 cells. Cells were transfected with control or SPOP-specific siRNAs for 48 hr followed by WB. (G) SPOP cannot induce polyubiquitination of hinge domain null AR splicing variants. 293T cells were transfected with the indicated plasmids for 16 hr followed by treatment with 20 µM MG132 for 8 hr, IP, and WB. (H) Differential effects of SPOP on the transcriptional activity of V7 hinge domain null and v567es variants of AR. 293T cells were transfected with PSA-Luc firefly luciferase reporter, renilla luciferase reporter, and the indicated plasmids. After 24 hr, cells were harvested for measurement of luciferase activities. Relative luciferase units (RLU) were determined by first normalizing the firefly units with the renilla activity and then normalized to the value of cells transfected with control vector (CV). All data shown are mean values ± SD (error bar) from three independent experiments. p < 0.01.

Increasing evidence suggests that C-terminal truncated AR splice variants play important roles in development of resistance to antiandrogen therapy in prostate cancer (Li et al., 2013; Sun et al., 2010). Because a majority of the AR splice variants identified thus far do not contain the hinge domain (Dehm and Tindall, 2011), where the SPOP-binding motif is located, we investigated whether AR splicing variants escape binding by SPOP. We demonstrated that the v567es variant, which harbors the SPOP-binding motif ^645^ASSTT^649^ (Figure 4A), binds to SPOP in a manner similar to AR WT (Figure 4B). None of the hinge domain null AR variants examined, including AR-V2, V5, V7, and V4 (Dehm and Tindall, 2011), was bound by SPOP (Figures 4A and 4B). Importantly, the steady-state levels of all of the AR variants examined, except v567es, were unaffected by SPOP (Figure 4C).

The hinge domain null AR variants V2, V5, V7, and V4 are predominantly expressed in 22Rv1 prostate cancer cells (Dehm et al., 2008; Guo et al., 2009; Hu et al., 2009). We demonstrated that SPOP binds to endogenous full-length AR, but not these endogenous variants in 22Rv1 cells (Figure 4D). Accordingly, overexpression of SPOP WT, but not the enzymatic dead mutant SPOPΔBTB, decreased the steady-state level of endogenous full-length AR in a dose-dependent manner. In contrast, SPOP WT had little or no effect on the level of endogenous AR variants in 22Rv1 cells (Figure 4E). Knockdown of endogenous SPOP increased the steady-state level of endogenous full-length AR but not the variants in 22Rv1 cells (Figure 4F). Furthermore, over-expression of SPOP increased polyubiquitination of AR WT and v567es, but not the hinge domain null variants in 293T cells (Figure 4G). Finally, we found that the hinge domain null AR variant AR-V7, but not the hinge domain-containing variant v567es, was resistant to SPOP-induced inhibition of its transcriptional activity (Figure 4H). Together, our data demonstrate that hinge domain null AR splicing variants are resistant to SPOP-mediated degradation. Thus, constitutive transcriptional activity of these variants is not affected by SPOP.

### Prostate-Cancer-Associated Mutants of SPOP Cannot Bind to and Promote AR Ubiquitination and Degradation

**Figure 5:**
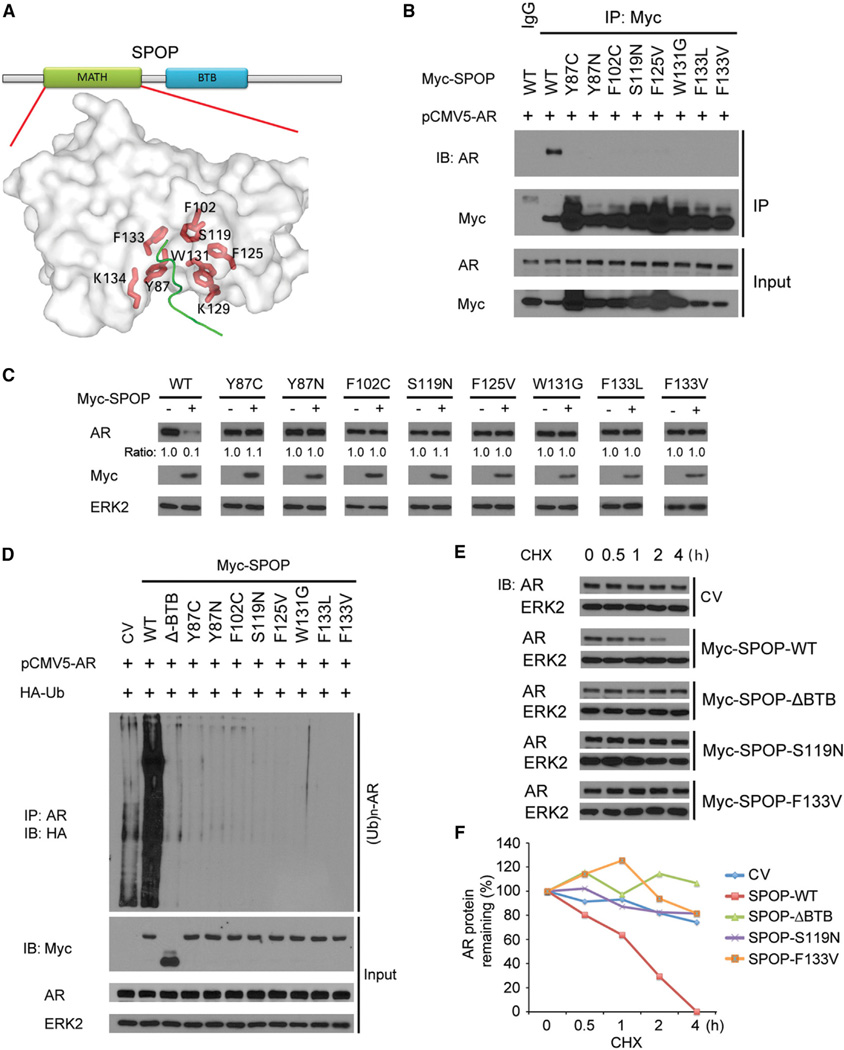
Prostate-Cancer-Associated Mutants of SPOP Fail to Promote AR Degradation (A) Computer modeling of the SPOP MATH domain indicating the positions of the residues mutated in prostate cancer. The side chains of mutated residues are shown as sticks in red; the substrate is shown as a tube in green; the MATH domain is shown as transparent surface in light gray. The figure is made using software vmd-1.9 (http://www.ks.uiuc.edu/Research/vmd/). (B) Prostate-cancer-associated mutants of SPOP cannot interact with AR. 293T cells were transfected with the indicated plasmids for 16 hr followed by treatment with 20 µM MG132 for 8 hr, coIP, and WB. (C) Prostate-cancer-associated mutants of SPOP fail to induce degradation of endogenous AR. C4-2 cells were transfected with wild-type (WT) or mutated SPOP as indicated for 24 hr followed by WB. The density of AR was determined by normalizing to ERK2 (loading control) first and then to the normalized value in Myc-SPOP-untransfected cells. (D) Prostate-cancer-associated mutants of SPOP cannot promote AR ubiquitination. 293T cells were transfected with the indicated plasmids. After 16 hr, cells were treated with MG132 for 8 hr, and cell lysates were prepared for IP and WB. (E and F) Prostate-cancer-associated mutants of SPOP have little or no effect on protein turnover of endogenous AR. LNCaP cells were transfected with control vector (CV), wild-type, or mutated SPOP. After 24 hr, cells were treated with 50 µg/ml cycloheximide (CHX), and at various time points cells were harvested for WB (E). At each time point, the intensity of AR was normalized to the intensity of ERK2 first and then to the value at the 0 hr time point (F). A similar result was obtained in two independent experiments.

Recent large-scale somatic mutation studies revealed that *SPOP* is one of the most frequently mutated genes in human prostate tumors (Barbieri et al., 2012; Kan et al., 2010). All the SPOP mutations found thus far occur in the MATH domain, a substrate-binding motif (Zhuang et al., 2009). Structural analysis of these sites has revealed that all the mutated residues are present on the surface of the substrate interaction pocket (Barbieri et al., 2012; Zhuang et al., 2009). We generated a series of Myc-tagged prostate-cancer-associated mutants of SPOP, including Y87C, Y87N, F102C, S119N, F125V, W131G, F133L, and F133V (Barbieri et al., 2012; Kan et al., 2010), and examined their interaction with AR. We demonstrated that, apart from SPOP WT, none of the mutants binds to AR (Figures 5A and 5B). Accordingly, all the mutants failed to affect the steady-state levels of endogenous AR in C4-2 cells (Figure 5C). None of the prostate-cancer-associated mutants were able to promote AR polyubiquitination (Figure 5D). Finally, we examined the effect of SPOP mutations on AR protein turnover using S119N and F133V mutants as two representatives (Barbieri et al., 2012). Similar to the enzymatic dead mutant SPOP-ΔBTB (positive control), S119N and F133V failed to accelerate AR protein turnover in comparison to SPOP WT (Figures 5E and 5F). These data suggest that prostate-cancer-associated mutations of SPOP abrogate its ability to bind and promote ubiquitination and degradation of AR.

### Androgens Attenuate SPOP-Mediated AR Degradation

**Figure 6:**
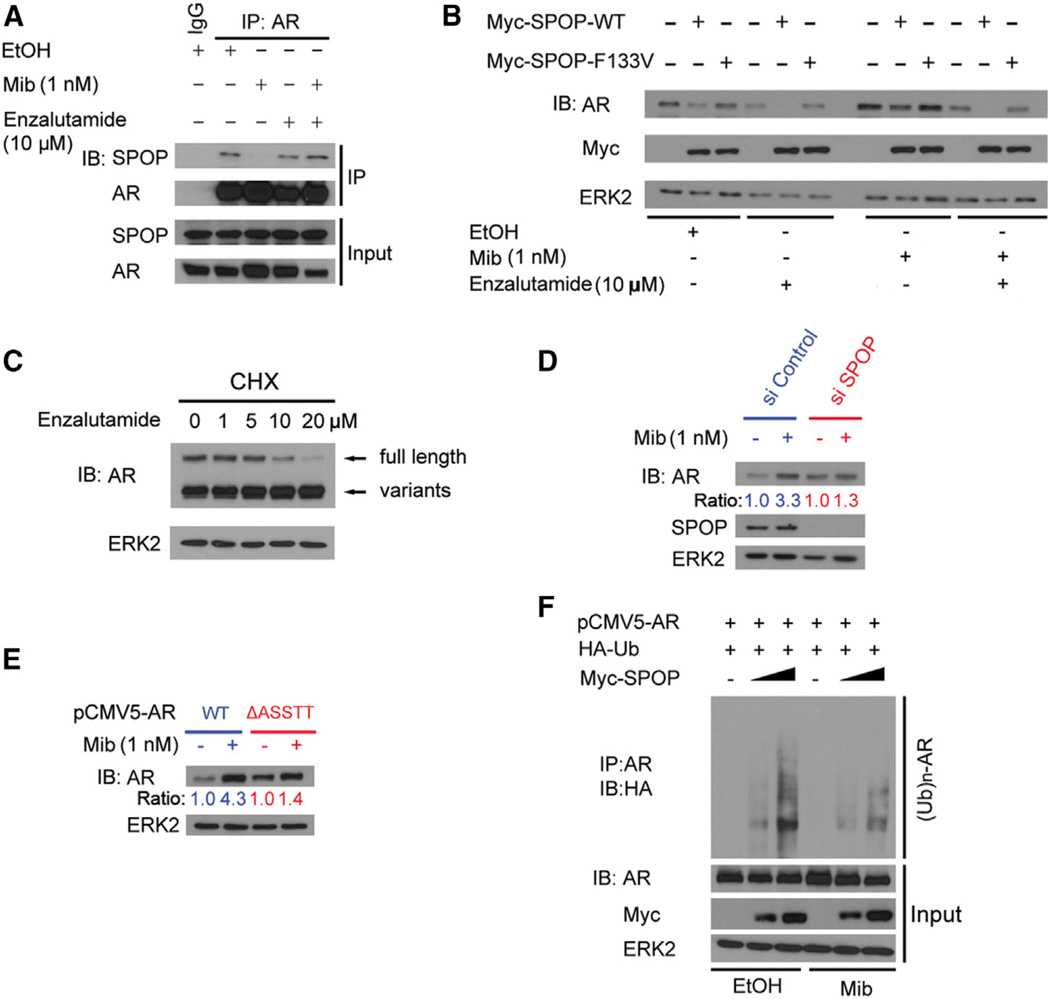
Androgens Attenuate SPOP-Mediated Degradation of AR (A) Androgens attenuate the SPOP-AR interaction, and this effect is blocked by the antiandrogen enzalutamide. C4-2 cells were cultured in androgen-depleted medium and treated with the vehicle ethanol (EtOH), 1 nM mibolerone (Mib), and/or 10 µM enzalutamide for 24 hr followed by MG132 treatment for 8 hr, coIP, and WB. (B) The antiandrogen enzalutamide enhances SPOP-mediated degradation of endogenous AR. C4-2 cells were transfected with the indicated plasmids and cultured in regular (androgen-containing) medium for 24 hr followed by treatment with mibolerone and/or enzalutamide for 24 hr and WB. (C) Enzalutamide induces degradation of endogenous full-length AR but not variants in 22Rv1 cells. Cells grown in regular (androgen-containing) medium were pretreated with 10 µg/µl cycloheximide (CHX) for 4 hr and then treated with different doses of enzalutamide for 20 hr followed by WB. (D) Knockdown of SPOP diminishes androgen-induced increase in AR protein levels. LNCaP cells were transfected with control or SPOP-specific siRNA and cultured in androgen-depleted medium for 24 hr. Cells were then treated with or without 1 nM mibolerone (Mib) for 24 hr followed by WB. The density of AR was determined by normalizing to ERK2 first and then to the normalized value in mock-treated cells. (E) Differential effects of androgens on the protein level of wild-type AR and the SPOP degradation-resistant mutant. 293T cells were transfected with the indicated plasmids and cultured in androgen-depleted medium for 24 hr. Cells were then treated with or without 1 nM mibolerone (Mib) for 24 hr followed by WB. The density of AR was determined by normalizing to ERK2 first and then to the normalized value in mock-treated cells. (F) Androgens attenuate SPOP-induced polyubiquitination of AR. 293T cells were transfected with the indicated plasmids (4 µg pCMV5-AR, 0, 2, or 4 µg Myc-SPOP) and treated with or without 1 nM mibolerone for 24 hr followed by treatment with MG132 for 8 hr, IP, and WB.

It has been known that androgens increase AR protein levels, whereas they decrease *AR* mRNA expression (Krongrad et al., 1991; Kumar et al., 1994). However, the molecular mechanism by which androgens upregulate AR protein is not fully under- stood. To determine whether SPOP plays a role in this process, we examined whether androgen treatment affects SPOP-AR interaction. As shown in Figure 6A, treatment of C4-2 cells with mibolerone, a synthetic androgen, largely diminished the interaction between endogenous AR and SPOP. This was completely reversed by treatment with the antiandrogen enzalutamide (Figure 6A). It is not surprising that enzalutamide treatment alone did not affect the AR-SPOP interaction because cells were cultured in androgen-depleted medium (Figure 6A). We further demonstrated that wild-type SPOP-induced downregulation of AR protein was largely inhibited by mibolerone, but markedly enhanced by enzalutamide in C4-2 cells cultured in regular (androgen-containing) medium (Figure 6B). In contrast, enzalutamide failed to enhance AR downregulation in C4-2 cells expressing the prostate-cancer-associated SPOP mutant F133V (Figure 6B). To further assess the effect of enzalutamide on AR degradation, we pretreated 22Rv1 cells with cycloheximide to block protein synthesis. Under this condition, we found that enzalutamide induced downregulation of full-length AR protein but not AR variants in a dose-dependent manner (Figure 6C). Furthermore, we demonstrated that knockdown of endogenous SPOP largely diminished androgen-induced upregulation of endogenous AR protein in LNCaP cells (Figure 6D). Next, we examined whether the androgenic effect on SPOP regulation of AR degradation is mediated through the degron motif ^645^ASSTT^649^. As shown in Figure 6E, mibolerone treatment significantly increased the protein level of wild-type AR in 293T cells, but this effect was largely diminished with the ΔASSTT mutant, which cannot bind to SPOP (Figure 3B). Finally, we demonstrated that androgen treatment decreased SPOP-mediated polyubiquitination of wild-type AR (Figure 6F). These data suggest that SPOP plays a critical role in androgen-induced stabilization, and antiandrogen-induced destabilization, of AR in prostate cancer cells.

## DISCUSSION

**Figure 7:**
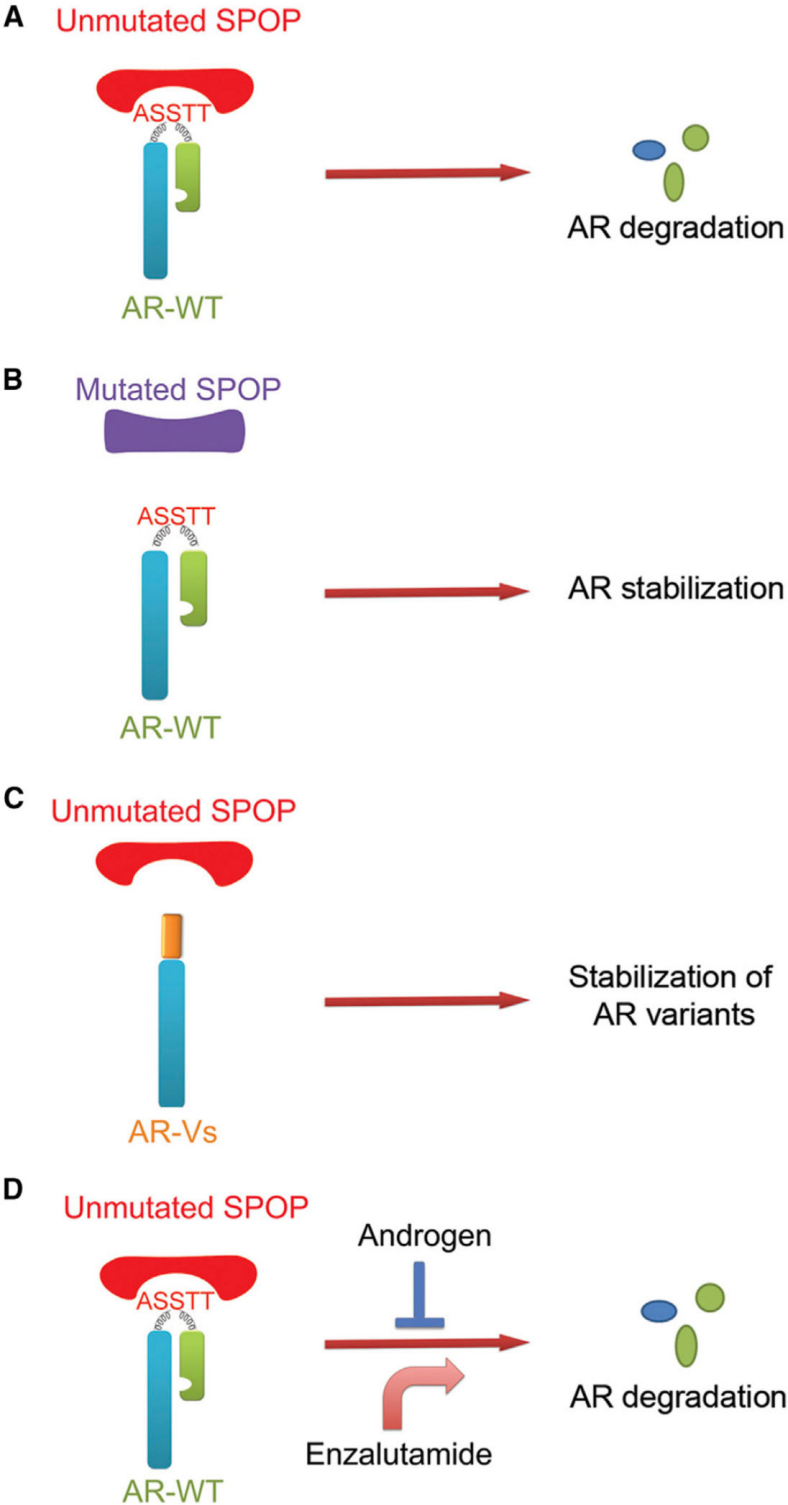
Models Depicting SPOP-Mediated Degradation of AR in Physiological and Pathological Conditions in Prostate Cancer (A) Unmutated SPOP promotes degradation of full-length wild-type AR (AR-WT). (B) Prostate-cancer-associated SPOP mutants lose the capacity to promote AR degradation. (C) Prostate-cancer-derived hinge domain-deficient AR splice variants escape from SPOP-mediated degradation. (D) Androgens attenuate SPOP-mediated degradation of AR, whereas the antiandrogen enzalutamide accelerates this process.

A large-scale somatic mutation study demonstrated that in approximately 450 tumors, comprising breast, lung, ovarian, pancreatic, and prostate cancer, the *SPOP* gene was highly mutated in prostate cancer (Kan et al., 2010). Recurrent *SPOP* mutations in prostate cancer have been confirmed by three independent genome-wide studies (Barbieri et al., 2012; Berger et al., 2011; Grasso et al., 2012). However, the pathophysiology of prevalent *SPOP* mutations in prostate cancer is poorly understood. In the present study, we provide evidence that all of the known prostate-cancer-associated mutants of SPOP lose their ability to bind to and promote AR ubiquitination and proteasome degradation. Reciprocally, all the hinge domain null AR splice variants escape SPOP-mediated degradation. We also provide evidence that androgens attenuate, whereas antiandrogens potentiate, SPOP-mediated degradation of AR. Thus, our study not only identifies AR as a bona fide ubiquitination and degradation substrate of SPOP, but also demonstrates that the effect of SPOP on AR degradation is subjected to regulation by physiological and pathological conditions in prostate cancer cells, including SPOP mutation, AR splicing, exposure to androgen, and antiandrogen treatment (Figure 7). It is worth noting that the *TMPRSS2-ETS* fusion genes, commonly detected in approx-imately 50% human prostate cancers, are positively regulated by AR (Kumar-Sinha et al., 2008; Tomlins et al., 2005). Intriguingly, SPOP mutations (function to stabilize AR) and TMPRSS2-ETS translocations are mutually exclusive in prostate cancer (Barbieri et al., 2012). A plausible explanation is that mutations in SPOP may reverse wild-type SPOP-mediated degradation of protein(s) that enable to inhibit TMPRSS2-ETS fusion formation, ETS activity, or both. Further investigation of this concept is warranted.

Activity and abundance of AR protein are crucial for prostate cancer cell proliferation, tumor progression, and development of resistance to antiandrogen therapies (Chen et al., 2004). AR protein levels and functions are subjected to regulation by various posttranslational modifications including phosphorylation, acetylation, methylation, SUMOylation, and ubiquitination (van der Steen et al., 2013). Several E3 ubiquitin ligases, including MDM2, CHIP, RNF6, and SIAH2, have been found to bind to AR and play important roles in either promoting AR degradation or activating AR under various cellular conditions (He et al., 2004; Lin et al., 2002; Qi et al., 2013; Xu et al., 2009). Our identification of SPOP as a bona fide E3 ligase of the AR is highly relevant to prostate cancer, because SPOP-mediated degradation of AR protein is disrupted by its mutations identified in prostate cancer, as well as the majority of prostate cancer-derived AR splicing variants.

SPOP substrates have a consensus motif Φ-π-S-S/T-S/T (Φ, nonpolar; π, polar) that mediates SPOP binding (Zhuang et al., 2009). This motif is invariably present in SPOP degradation targets, including the phosphatase Puc (Liu et al., 2009), the transcription regulators Ci/Gli and Daxx (Kwon et al., 2006; Zhang et al., 2006), and the chromatin component MacroH2A (Hernán-dez-Muñoz et al., 2005). Through motif analysis, we identified a perfectly matched SPOP-binding motif (^645^ASSTT^649^) in AR. Deletion of the ^645^ASSTT^649^ motif completely abolished the interaction between SPOP and AR, as well as SPOP-mediated polyubiquitination of AR. Thus, like other canonical SPOP substrates, AR contains a functional SPOP-binding consensus motif. SRC-3, a coactivator of AR that is often upregulated in prostate cancer (Zhou et al., 2005), is another SPOP ubiquitination target (Geng et al., 2013; Li et al., 2011). A similar sequence is present in SRC-3 upon phosphorylation at serine 102 by casein kinase IE (Li et al., 2011). Importantly, prostate-cancer-associated mutants of SPOP lose the capability to degrade SRC-3 (Geng et al., 2013). Thus, it is possible that SPOP mutations augment AR functions in prostate cancer by inhibiting turnover of both AR and its coactivator SRC-3.

A number of AR splice variants have been identified (Dehm and Tindall, 2011). Increasing evidence suggests that expression of AR variants is upregulated during castration-resistant progression of prostate cancer and that increased expression of AR variants may contribute to resistance to antiandrogen therapies (Hörnberg et al., 2011; Hu et al., 2012; Li et al., 2013; Sun et al., 2010; Watson et al., 2010). In agreement with our finding that the SPOP-binding motif ^645^ASSTT^649^ is located in the hinge domain of the AR, we demonstrated that none of the hinge domain null AR variants could be bound by SPOP, thereby escaping SPOP-mediated protein degradation in prostate cancer cells. In contrast, we showed that the v567es variant (also known as AR-V12), in which the hinge domain remains, can be bound and degraded by SPOP. Because the majority of the AR variants identified thus far do not harbor the hinge domain (Dehm and Tindall, 2011), AR splicing represents a key mechanism by which the AR protein escapes SPOP-mediated polyubiquitination and proteasome degradation, thereby contributing to prostate cancer pathogenesis. Expression of AR v567es variant increases the stability of full-length AR protein without increasing mRNA levels in LNCaP cells (Sun et al., 2010). Thus, it is worthwhile to investigate in the future whether full-length AR and splicing variants can also be differentially targeted by other E3 ubiquitin ligases for degradation. Moreover, we provide evidence that the antiandrogen enzalutamide enhances degradation of full-length AR but has no effect on AR variants in 22Rv1 cells. Thus, escaping of AR variants from SPOP-mediated proteolysis may represent an important mechanism that confers resistance to antiandrogen therapy.

Another important finding of our study is that androgen attenuates SPOP binding and degradation of AR, whereas enzalutamide promotes this process. Such a dynamic regulation of the AR thus identifies a paradigm for drug design. Enzalutamide is an antiandrogen that inhibits the transcriptional activity of AR by competitively blocking the binding of androgens to AR. Intriguingly, we found that this drug induces AR degradation by facilitating SPOP-AR interaction. One possible explanation for this is that, when androgens bind to AR, AR undergoes a conformation change, thereby affecting the binding of the ^645^ASSTT^649^ motif to SPOP and subsequent AR degradation. In contrast, when enzalutamide binds to the AR LBD domain, it blocks androgen binding and thereby facilitates SPOP-AR interaction and AR degradation. Such a concept may lead to novel strategies for developing drugs that can alter AR conformation, thus facilitating E3 ligase-mediated destruction of AR in prostate cancer.

In summary, we have identified AR as a substrate of SPOP. AR harbors a functional SPOP-binding consensus motif in the hinge domain. We demonstrated that SPOP promotes AR degradation and inhibits the transcriptional activity of the AR and AR-mediated prostate cancer proliferation. In contrast, the majority of AR variants can escape SPOP-mediated destruction due to their lacking of the hinge domain. Moreover, all SPOP mutations identified in human prostate cancers disrupt the SPOP-AR interaction and abolish SPOP-induced AR degradation. Additionally, we showed that the antiandrogen enzalutamide enhances SPOP-mediated degradation of full-length AR, but not most AR variants in prostate cancer cells. Given the importance of AR in prostate cancer initiation, progression, and therapy resistance, identification of AR as a substrate of SPOP E3 ligase provides a plausible explanation for the high frequency of SPOP mutations in prostate cancer. These findings also support a tumor-suppressor function of SPOP in prostate cancer and emphasize the importance of this pathway in development of resistance to antiandrogen therapy of prostate cancer in the clinic.

## EXPERIMENTAL PROCEDURES

For further details, see the Supplemental Experimental Procedures.

### Plasmids and Reagents

The mammalian expression vectors for HA-tagged ubiquitin (HA-Ub), wild-type AR (AR-WT), and AR variants V2, V4, V5, and V7 were described previously (Bohrer et al., 2013; Huang et al., 2005; Liu et al., 2008). The AR variant v567es was kindly provided by Dr. Donald Tindall at Mayo Clinic. HA-SPOP and Myc-SPOP were cloned into pCMV vector. AR mutants S646A, S647A, T648A, and T649A and prostate-cancer-associated mutants of SPOP were generated by site-specific mutagenesis (Stratagene). AR deletion mutants ΔEGSSS, ΔASSTT, 2Δ, and Myc-SPOP-ΔBTB were generated by KOD-Plus Mutagenesis Kit (Toyobo). Antibodies used were anti-AR (N20), anti-ERK2, anti-Myc tag (Santa Cruz Biotechnology), anti-SPOP (Abcam), and anti-HA (Covance). MG132, cycloheximide, and mibolerone were purchased from Sigma-Aldrich. The antiandrogen enzalutamide was kindly provided by Medivation.

### Immunoprecipitation and Western Blotting

Immunoprecipitations were performed as described previously (Huang et al., 2006; Wang et al., 2013). For western blotting, protein samples were prepared in modified RIPA buffer (1 × PBS, 1% NP-40, 0.1% SDS, and 1% protease inhibitor cocktails). Equal amounts of protein (50–100 µg) from cell lysates were denatured and subjected to SDS-PAGE and were transferred to nitrocellulose membranes (Bio-Rad). The membranes were immunoblotted with specific primary antibodies and horseradish-peroxidase-conjugated secondary antibodies and visualized by SuperSignal West Pico Stable Peroxide Solution (Thermo Scientific).

### Statistical Analysis

Experiments were carried out with three or more replicates unless otherwise stated. Statistical analyses were performed by Student’s t test for most studies. Values with p < 0.05 are considered statistically significant.

## Supplementary Material

Supplement I
