# Supplementary material for: Destruction of Full-Length Androgen Receptor by Wild-Type SPOP, but Not Prostate-Cancer-Associated Mutants
Source: Cell Rep. Author manuscript; Available in PMC 2015 Mar 17. (PMC4361392; doi:10.1016/j.celrep.2014.01.013)
Supplement: Supplement I [file NIHMS661448-supplement-Supplement_I.pdf]

Supplemental Information

Destruction of full-length androgen receptor by wild-type SPOP but not prostate cancer-associated mutants

Jian An, Chenji Wang, Yibin Deng, Long Yu, and Haojie Huang

SUPPLEMENTAL EXPERIMENTAL PROCEDURES

Cell lines, cell culture, cell transfection and luciferase reporter assay

The prostate cancer cell lines 22Rv1 and LNCaP and human embryonic kidney cell line 293T cells were purchased from ATCC (Manassas, VA). 22Rv1 and LNCaP cells were cultured in RPMI 1640 medium (Invitrogen) supplemented with 10% FBS (Hyclone). 293T cells were maintained in Dulbecco's modified Eagle's medium (Invitrogen) supplemented with 10% FBS. The C4-2 cell line was purchased from UroCorporation and grown in RPMI 1640 supplemented with 10% FBS. Cells were cultured at 37°C supplied with 5% CO_2. Transfections were performed by electroporation using an Electro Square Porator ECM 830 (BTX) (Chen et al., 2010) or by using Lipofectamine 2000 (Invitrogen). Approximately 75–90% transfection efficiencies were routinely achieved. For luciferase reporter assays, cells were harvested 24 h after transfection, and firefly and renilla luciferase activities in cell lysates were measured using a dual luciferase kit (Promega). Renilla luciferase activities of cells were used as internal controls.

RNA interference

Nonspecific control siRNA and siRNAs for human SPOP were purchased from Thermo Scientific Dharmacon. siRNAs for human RBX1 and CULLIN3 were

purchased from GenePharma. siRNA transfection of cells was performed following the manufacturer's instruction. siRNA sequence information is provided in Supplementary Table S1.

RT-qPCR

Total RNA was isolated from cells and cDNA was synthesized using the Super-Script kit from Invitrogen. Two-step real-time polymerase chain reaction (PCR) was performed using the SYBR Green Mix (BioRad) and an iCycler iQTM detection system (BioRad) according to manufacturer's instructions. Both forward and reverse primers were used at a final concentration of 200 nM. The expression of *GAPDH* gene in each sample was used as an internal control. Information for primers used is provided in Supplementary Table S1.

Glutathione S-transferase (GST) pull-down assay

293T cells were lysed 36 h after transfection with cell lysis buffer (20 mM Tris-HCl, pH 7.5, 150 mM NaCl, 0.1% Nonidet P40, 1 mM DTT (dithiothreitol), 10% glycerol, 1 mM EDTA, 2.5 mM $MgCl_2$ and 1 μg/ml leupeptin) for 30 min at 4°C. GST fusion proteins were immobilized on glutathione-Sepharose beads (GE Healthcare Lifescience). After washing with lysis buffer, the beads were incubated with lysates of transfected 293T cells for 4 h. The beads were then washed four times with binding buffer and resuspended in sample buffer. The bound proteins were subjected to SDS/PAGE.

MTS assay

Cell growth was monitored by absorbance using the MTS assay according to manufacturer's instructions (Promega). Briefly, cells were plated in 96-well plates at a density of 1,000 cells per well and cultured in androgen-depleted (charcoal-treated) medium. At the indicated times, 20 µl of CellTiter 96R AQueous One Solution Reagent (Promega) was added to cells. After incubating for 60 min at 37°C in the cell incubator, cell growth was measured in a microplate reader at 490 nm.

SUPPLEMENTAL REFERENCES

Chen, S., Bohrer, L. R., Rai, A. N., Pan, Y., Gan, L., Zhou, X., Bagchi, A., Simon, J. A., and Huang, H. (2010). Cyclin-dependent kinases regulate epigenetic gene silencing through phosphorylation of EZH2. Nat. Cell Biol. *12*, 1108-1114.

SUPPLEMENTAL TABLE

Table S1. RT-qPCR primer and siRNA sequences

Primer sequences

Gene	RT Forward	RT Reverse
AR	GTGGAAGCTGCAAGGTCTTC	TTCAGATTACCAAGTTTCTTCAGC
SPOP	AGCAAATGATAAACTGAAAT	GTCATCAGGGAGAAGCCCGT
PSA	AGGCCTTCCCTGTACACCAA	GTCTTGGCCTGGTCATTTCC
TMPRSS2	CTGCCAAGGTGCTTCTCATT	CTGTCACCCTGGCAAGAATC
GAPDH	GAAGGTGAAGGTCGGAGTC	GAAGATGGTGATGGGATTTC

siRNA Sequences

Gene	Sequence
si SPOP#1	5′-GGGAUACCAUGCAACAUAA-3′
si SPOP#2	5′-CUAGAAAUCUGUUGCUAUG-3′
si CUL3#1	5′-AACAACUUUCUUCAAACGCUA-3′
si CUL3#2	5′-AACAACACUUGGCAAGGAGAC-3′
si RBX#1	5′-GGGAUAUUGUGGUUGAUAA-3′
si RBX#2	5′-GGAACCACAUUAUGGAUCU-3′
